# Insights on the Use of Carbon Additives as Promoters of the Visible-Light Photocatalytic Activity of Bi_2_WO_6_

**DOI:** 10.3390/ma12030385

**Published:** 2019-01-26

**Authors:** Alicia Gomis-Berenguer, Irma Eliani, Vânia F. Lourenço, Rocio J. Carmona, Leticia F. Velasco, Conchi O. Ania

**Affiliations:** 1 POR2E group, CEMHTI, CNRS (UPR 3079), Université d’Orléans, 45071 Orléans, France; irmavaniaberliani@gmail.com (I.E.); vania.lourenco@cnrs-orleans.fr (V.F.L.); 2 Instituto Nacional del Carbón (INCAR-CSIC), Apdo. 73, 33080 Oviedo, Spain; rociojimenezcarmona@gmail.com; 3 Department of Chemistry, Royal Military Academy, Renaissancelaan 30, 1000 Brussels, Belgium; leticia.fernandezvelasco@rma.ac.be

**Keywords:** simulated solar light, bismuth tungstate, carbon materials

## Abstract

We have explored the impact of the incorporation of various amounts of carbons from varied physicochemical features as additives to Bi_2_WO_6_ for the photocatalytic degradation of a dye using simulated solar light. Data has revealed that the composition and acidic character of the carbon additive are important parameters in the performance of the Bi_2_WO_6_/carbon catalysts. The presence of a carbon additive improved the conversion of the dye, evidencing the occurrence of charge transfer reactions that involve radical mediated reactions. The catalysts prepared with 2 and 5 wt.% of carbon additive outperformed the bare semiconductor, despite the shielding effect of the carbon matrix. The acidic nature of the Bi_2_WO_6_/carbon catalysts governs the degradation pathway (due to the preferential adsorption of the dye), that proceeds via the deethylation of the auxochrome groups of the dye at short irradiation times, followed by the cleavage of the chromophore at long irradiation times. Regarding the characteristics of the carbons, the photocatalytic degradation rate is accelerated by carbons of acidic character and high oxygen content, whereas the porosity seems to play a minor role. The presence of the carbon additives also affects the toxicity of the treated solutions, rendering lower values after shorter irradiation periods.

## 1. Introduction

After the demonstration of the photocatalytic activity of ZnO and TiO_2_ electrodes in the 1960′s and 70’s [1,2], heterogeneous semiconductor photocatalysis has become a largely investigated topic, particularly in the field of energy conversion for environmental remediation. Although the photocatalytic activity of many transition metal oxides and sulfides has been widely investigated, TiO_2_ is considered the benchmark semiconductor for the degradation of pollutants in water, due to its low cost and large availability, chemical inertness, low toxicity, and stability against photo- and chemical corrosion [3,4]. However, it still has important limitations that prevent the large market integration of photocatalytic advanced oxidation processes based on TiO_2_ for water treatment (currently restricted to low effluent volumes and low pollutant concentration), mainly attributed to its low photonic efficiency under sunlight, a high (and pH-dependent) recombination rate of charge carriers, and a low to moderate surface area of most TiO_2_-based photocatalysts [3].

Numerous efforts have been made in the last decades to overcome this, such as semiconductor engineering synthesis (e.g., lattice doping, nanosizing catalysts, novel materials) aiming at band-gap tuning and reduction of the surface recombination [3,5,6,7,8,9,10,11]. Among different photoactive materials, bismuth-based semiconductors offer interesting perspectives as alternatives to TiO_2_ in sunlight-driven photocatalysis due to their good light absorption properties under visible light [12,13,14]. However, these materials display a weak stability during long illumination periods, and very often their optical and physical features (surface area, morphology, particle size, crystallinity) are strongly affected by the synthesis method [15,16]. All of this affects their photoactivity and constitutes a challenge for efficiently exploiting their potentialities for an upscale of the photocatalytic process.

On the other hand, the use of carbon either as semiconductor dopant or as additive has been revealed as an interesting alternative to attain high photoconversion efficiencies of various semiconductors, due to its ability to delocalize the charge carriers (thereby reducing surface recombination) in the π-electron density of the graphitic layers [17,18]. Nanostructured carbons such as graphene or carbon nanotubes have been extensively investigated as additives to semiconductors due to their high electron mobility [19,20]. Various forms of porous carbons have also been used for this purpose, due to the increased mass transfer upon adsorption of the pollutant on the porosity [21,22]. In this regard, recent investigations including our own have reported that the photocatalytic performance of Bi_2_WO_6_ can be enhanced by the use of certain amounts of carbon additives; nevertheless, the effect of the carbon additive has been barely addressed, and the optimum nominal carbon content seems to be unclear and dependent on the nature of the carbon additive [23,24,25,26,27].

Taking this into account, the objective of this work was to throw some light on the impact of the physicochemical features of carbon additives on the photocatalytic performance of Bi_2_WO_6_/carbon photocatalysts, using carbon materials of various origins and characteristics, covering composition, acidity, and porosity. As the target compound, we have used Rhodamine B, a toxic fluorophore widely used in industrial purposes such as dyeing in textiles, paper, and paints, cosmetic products, and imaging in biotechnology applications [28]. This recalcitrant dye is frequently found in wastewater, thus its photocatalytic degradation has been extensively reported. Our results provide new insights on the photodegradation mechanism of a dye using Bi_2_WO_6_/carbon catalysts, and demonstrate the correlation between the photocatalytic activity of the hybrid semiconductor/carbon catalyst and the origin and the amount of the carbon additive, specific surface interactions and nanopore confinement.

## 2. Materials and Methods

### 2.1. Materials

Several carbon materials with varied physicochemical and structural characteristics were selected in this study: sample CL prepared by chemical activation in H_3_PO_4_ (ca. 0.5 g P/g) of a coconut shell at 450 °C; sample CB, obtained upon steam activation (ca. 850 °C) of bituminous coal (supplier Agrovin SA, Alcázar de San Juan, Spain); sample CS, obtained by hydrothermal carbonization of 0.5 M glucose at 180 °C (heating rate of 100 °C/h) for 10 h [29]. Before use, all of the carbon materials were washed in distilled water to eliminate the excess of reactants and other impurities, and stored in a desiccator.

For the preparation of the photocatalysts, the semiconductor Bi_2_WO_6_ (BWO) was synthesized by a hydrothermal treatment in the presence of different amounts of the carbon materials ranging from 2–5 wt.% [27]. Briefly, about 0.01 mol of Bi(NO_3_) _3_·5H_2_O (Sigma Aldrich, St. Louis, MO, USA, purity ≥98.0%) were dissolved in 10 mL of glacial acetic acid and 0.005 mol of Na_2_WO_4_·2H_2_O (Sigma Aldrich, purity ≥99%) were dissolved in 90 mL of distilled water. Both solutions were mixed with the adequate amount of carbon additive, sonicated for 15 min and stirred for 30 min to allow a good dispersion of the reactants. The suspensions were then transferred into a Teflon-lined stainless steel autoclave and heated at 140 °C for 20 h. The obtained solids were filtered, repeatedly washed and dried overnight at 120 °C. The control synthesis of the bare semiconductor (sample BWO) in the absence of carbon additives was carried out in the same conditions. The photocatalysts were labeled as BWO/X-Y, X being the acronym of the carbon material and Y the amount (wt.%) incorporated.

### 2.2. Characterization Techniques

The porosity of the catalysts and the carbon materials was determined by N_2_ adsorption/desorption isotherms at 77 K in a volumetric analyzer (Micromeritics, Norcross, GA, USA). The samples were previously outgassed under vacuum at 120 °C for 17 h. The gas adsorption isotherms were used to calculate the specific surface area (S_BET_), total pore volume (V_PORES_), and micropore volume (W_0_), the latter using the Dubinin-Radushkevich equation [30]. The morphology and microstructure of the catalysts were characterized by scanning electron microscopy, recorded using a FE-SEM apparatus (QuantaSEM, FEI, Hillsboro, OR, USA) operating at 24 kV. The crystalline phase composition was estimated by X-ray diffraction (XRD), using a Bruker instrument (D8 Advance, Manning Park Billerica, MA, USA) operating at 40 kV and 40 mA and using CuKα (0.15406 nm) radiation. The optical features of the photocatalysts were determined by UV-Vis diffuse reflectance spectroscopy (Shimadzu UV-2501, Kyoto, Japan) in a spectrophotometer equipped with an integrating sphere and using BaSO_4_ as a blank reference. Measurements were recorded between 220–700 nm (due to the configuration of the integrating sphere) in the diffuse reflectance mode and transformed to a magnitude proportional to the extinction coefficient through the Kubelka-Munk function, F(R∞) for the estimation of the energy band gap. The acidity of the catalysts was characterized by NH_3_ chemisorption in a volumetric analyzer (Micromeritics). About 400 mg of the catalysts were previously outgassed at 120 °C; an adsorption isotherm of ammonia at 40 °C was recorded from vacuum up to 500 mm Hg. Subsequently, the physisorbed fraction was removed by heating at 40 °C for 4 h, and a second NH_3_ isotherm was recorded at the same temperature. The amount of chemisorbed ammonia corresponds to the difference in the volume adsorbed from the first and second isotherm, and was used to estimate the total amount of acid sites of the catalysts [31]. The hydrophobic/hydrophilic nature of the catalysts was characterized by the determination of the surface pH of an aqueous suspension (ca. 1 g/L) of the catalysts. Elemental analysis of the carbon materials was measured in a LECO CHNS-932, Saint Joseph, MI, USA (carbon, hydrogen, nitrogen, and sulfur content) and LECO VTF-900 (oxygen content) automatic analyzers. The transfer of the photogenerated charge carriers in the catalysts was studied by electron spin resonance spectroscopy (ESR) using a nitrone spin trapping agent (5,5-dimethylpyrroline-N-oxide, DMPO). This compound is capable of forming spin adducts [32] with hydroxyl and superoxide radicals formed when the charge carriers react with the aqueous solution. Briefly, 0.5 g/L of the catalysts were suspended in 5 ml of HClO_4_ buffer (pH 3) and the appropriate volume of DMPO to reach 18 mM. Samples were irradiated for 20 min (Philips, Amsterdam, Netherlands, TL K40W/05 lamp, emission peak centered at 365 nm). ESR spectra were recorded from the solution at room temperature on a Bruker ESP 300E X band spectrometer [33].

### 2.3. Photocatalytic Performance

Photodegradation experiments were performed at room temperature using simulated solar light (Osram Ultra-Vitalux lamp, 300 W) with a photon flux of ca. 120 mW/m^2^ as determined by a photodiode (Thorlabs, Newton, NJ, USA). The emission spectrum of the lamp is shown in Figure S1 in the Supporting Information. A batch reactor (250 mL) was employed with a catalysts loading of 1 g/L, and an air flow to assure excess of oxygen during the photocatalytic runs [27]. The suspensions were equilibrated under stirring in the dark for 60 min (until no further adsorption took place). Due to the porous character of the photocatalysts, the initial concentration of Rhodamine B (RhB) in the solution was adjusted for each catalyst (based on the amount adsorbed) to obtain a concentration of ca. 10 ppm when the illumination was applied. The initial pH of the suspensions was ca. 5 for all the catalysts, and remained unchanged during the photocatalytic assays. The photooxidation of the dye was followed by UV-Vis spectrophotometry and HPLC (C18 column, mobile phase 5 mM H_2_SO_4_:CH_3_OH of 35:65 v/v, flow rate of 0.8 mL/min, injection volume 50 μL). Aliquots of ca. 2 mL were removed periodically during the experiments and filtered (0.45 mm nylon filter) before measurement; Total Organic Carbon (TOC) was followed also by means of a TOC analyzer (Shimadzu VCPH). The corresponding control experiments (photolysis) in the absence of photocatalysts were also conducted in similar conditions, for comparison purposes. All of the experiments were performed at least in duplicate, with an accuracy of ca. 5% for the photocatalytic runs (average data is presented).

### 2.4. Toxicity Measurements

The toxicity of the solution after the photocatalytic treatment at various sunlight irradiation times was evaluated by the luminescence inhibition of *Vibrio Fisheri* test, using a Microtox M500 Analyzer (London, UK) according to international procedures (ISO 11348-3, ASTM D5660). The bioluminescent bacteria were provided by Hach Lange France SAS. The luminescence intensity of *V. Fischeri* bacteria was measured after 5 and 15 min of exposition to the solutions at 15 °C, under 20% NaCl and adjusting the pH to 6–8 when necessary. All of the solutions were previously filtered (0.45 mm nylon filter) to prevent contact of the catalyst powders with the bacteria. Sensitivity of the bacteria was previously tested by using reference compounds (phenol and zinc sulphate). Results were used to determine the luminescence inhibition after 15 min of exposure (%) at dilution rates between 50–5%. All of the tests were performed at least in duplicates for all of the dilutions and exposure times.

## 3. Results and Discussion

Various studies, including our own, have reported that the photocatalytic activity of certain semiconductors (including Bi_2_WO_6_) can be improved through the incorporation of a carbon additive in the formulation of the photocatalyst. However, neither the optimum nominal carbon content nor the role of the nature of the carbon additive on such improved performance seem to be well understood [23,24,25,26,27]. Aiming to clarify the impact of the physicochemical features of the carbon material used as additive, we have studied the photocatalytic activity of Bi_2_WO_6_/carbon catalysts containing low amounts of carbon additive and using carbon materials of varied origin and properties (covering composition, acidity and porosity).

### 3.1. Characterization of the Photocatalysts

**Figure 1:**
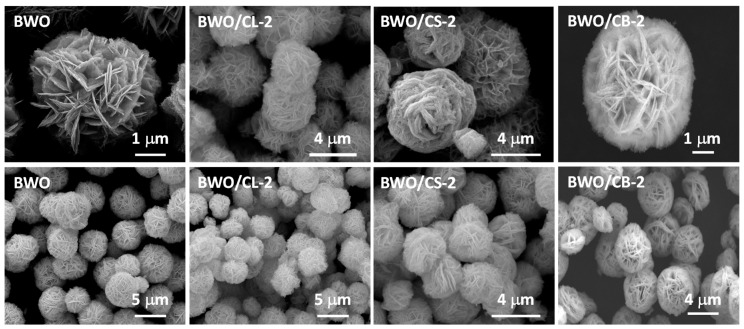
SEM images of Bi_2_WO_6_ (BWO) and the hybrid photocatalysts containing 2 wt.% of carbon additive.

**Figure 2:**
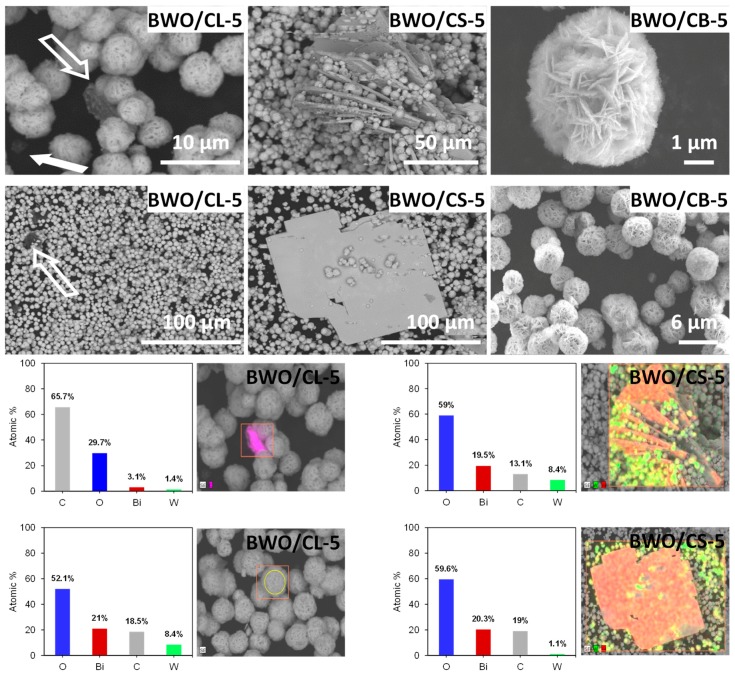
Selected SEM images of samples BWO/CL-5, BWO/CS-5, BWO/CB-5, arrows indicate the presence of a new phase. The EDX spectra from different regions marked with a square.

Figure 1 and Figure 2 show selected SEM images of the studied catalysts. As seen, bismuth tungstate nanostructures are formed by 3D flower-like shaped nanoparticles with particles sizes ranging from 3 to 9 μm, as reported in the literature [23,34]. The incorporation of the carbon additive in the hydrothermal synthesis barely modified the morphology of the semiconductor nanoparticles, although the average size was slightly reduced to ca. 1–3 μm. For the catalysts incorporating 2 wt.% of carbon (Figure 1), it was not possible to detect isolated carbon particles (average particle size of ca. 200 nm for CS, and ca. 1–5 μm for CL and CB, see Figure S2), confirming a good dispersion of the carbon materials within the particles of the semiconductor. Some catalysts containing 5 wt.% carbon additive (e.g., BWO/CL-5) showed an opposed behavior.

For samples BWO/CL-5 and BWO/CS-5, some plate-shaped aggregates of a few microns length were observed within the BWO particles (Figure 2). EDX measurements conducted to identify the composition of this new phase indicated a high content of Bi and O elements in these aggregates, with almost no traces of W or carbon (Figure 2). We have assigned them to Bi_2_O_3_ or BiOOH nanostructures. These aggregates were not observed for BWO or the catalysts with 2 wt.% of carbon, and thus are attributed to the presence of high amounts of carbon additive during the hydrothermal synthesis of the BWO/carbon photocatalysts. Interestingly, the formation of such plate-shaped aggregates of BWO was only observed for the catalysts incorporating the carbon additives of an acidic nature (Table 1), indicating that the acidity of the materials is important for the hydrothermal reaction. Indeed, it has been reported that Bi_2_O_3_ polymorphs of varied morphology can be formed at hydrothermal conditions following a pH-sensitive reaction [35,36]. These changes in the morphology are expected to influence the photocatalytic activity of the samples, as it will be discussed below.

The crystalline structure of the semiconductor corresponds to the orthorhombic phase of the semiconductor (JCPDS 39-0256) [37]; the BWO/carbon photocatalysts displayed similar XRD patterns (Figure S2) that the pristine semiconductor, regardless the nature and nominal content of the carbon additive. None of the reflections of the carbon additives (e.g., the broad peaks at 002 and 10 characteristic of carbons with a turbostratic structure) were observed in the XRD patterns of the BWO/carbon catalysts (Figure S3); this is most likely attributed to the low amount of carbon in their composition, and low intensity of the reflections of the carbons, compared to the peaks of the semiconductor. The lack of diffraction peaks assigned to bismuth and tungsten oxides confirmed the complete self-assembling of the BWO precursors when the hydrothermal synthesis was carried out in the presence of the carbon additives.

**Figure 3:**
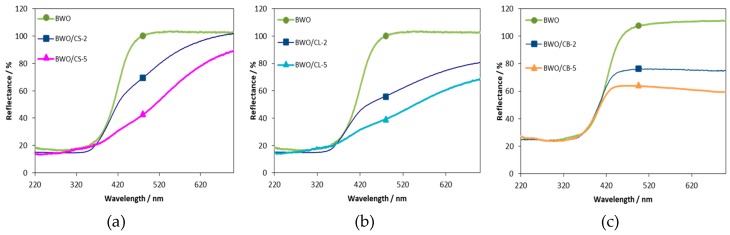
Diffuse reflectance spectra of Bi_2_WO_6_ and the photocatalysts incorporating various amounts of the (**a**) CS, (**b**) CL and (**c**) CB carbon additive.

The carbon additive induced some changes in the optical response of the BWO/carbon catalysts, as seen in UV-visible diffuse reflectance spectra (Figure 3). The spectrum of BWO presented the characteristic absorption sharp edge above 400 nm due to the transitions from the O2p and Bi6s hybrid orbitals to the W5d and Bi6p orbitals, and yielding a band gap of ca. 2.8 eV, as reported in the literature [12]. The spectra of the BWO/carbon catalysts showed a marked decrease in the reflectance at high wavelengths, attributed to the absorbance of the carbon additives. This reduced reflectivity was more pronounced with the amount of carbon additive, and is attributed to the shielding effect of the carbon matrix. Furthermore, in the case of samples BWO/CS and BWO/CL (for both 2 and 5wt.%) the spectra show a curvature between 445–700 nm with a smooth slope increasing up to maximum reflectance, as opposed to the sharp absorption cut-off of BWO and BWO/CB samples. This has been attributed to the absorption of photo-sensitive groups on the carbon matrix [38,39], and is supported by the high oxygen content of carbon CL and CS, as opposed to carbon CB (Table 1). Furthermore, the absorption edge corresponding to Bi_2_O_3_ (ca. 420 nm) [40,41] was not observed for any of the catalysts, suggesting the particles of bismuth oxide detected by SEM in samples BWO/CL-5 and BWO/CS-5 are not optically active.

Concerning porosity, the main textural parameters of the catalysts are compiled in Table 1. Data corresponding with the carbon materials used as additives are also included for clarity. All the catalysts displayed type II adsorption isotherms (Figure S4) characteristic of materials with a low porous development, with a marked hysteresis loop at relative pressures above 0.8. Given the morphology of the particles, this feature is associated with gas condensation in the voids of the petals of the flower-like particles. Similar textural features have been reported for this semiconductor [23]. The incorporation of the carbon additive provoked a subtle increase in the micropore volume (Table 1, Figure S4). This effect was more pronounced for the catalyst prepared with carbons CL and CB, both displaying a more microporous character. Interestingly, the nanoporous texture of the catalysts did not follow the expected trend based on stoichiometry considering the loading of the carbon and the porosity of both phases. Since the surface area and pore volume are extensive magnitudes, this observation suggests that the carbon additive is intimately interacting with the semiconductor particles, likely inside the layered structure of WO_6_ octahedra and (Bi_2_O_2_)^2+^ units, rather than existing as isolated particles that would otherwise render the stoichiometric textural features of a physical mixture of the components of the catalyst.

### 3.2. Photocatalytic Degradation of RhB

**Figure 4:**
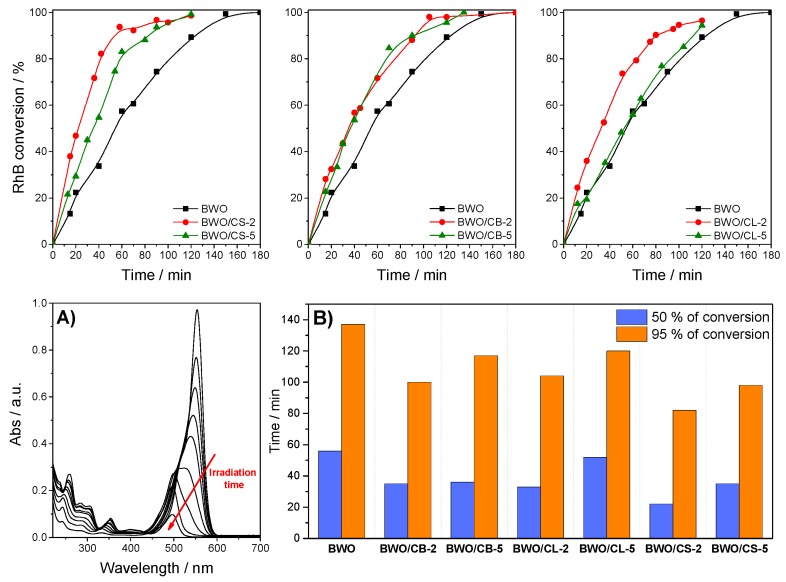
(first row) Rhodamine B conversion of studied photocatalysts after irradiation using simulated solar light; (second row) (**A**) Example of the evolution of the UV-vis spectra of the aqueous solutions of RhB upon irradiation of BWO/CB-2 catalyst with the irradiation time; (**B**) Time needed to reach 50 and 85% of Rhodamine B conversion.

Figure 4 shows the photocatalytic performance of the series of Bi_2_WO_6_/carbon catalysts towards the degradation of RhB under simulated solar light, in terms of RhB conversion. The conversion corresponding to the photolytic degradation in the absence of any catalyst was around 5% after 3 h of irradiation (data is compiled in Table S1). As indicated in the experimental section, it is important to point out that all the photocatalytic runs were carried out using the same initial concentration of the dye in a solution at the beginning of the irradiation; this is important when dealing with porous catalysts to discriminate the photocatalytic reaction from the fraction adsorbed in the pores (i.e., the impact of adsorption in the overall kinetics and conversion).

As seen, the conversion of RhB after 2 h of irradiation was close to 100% for all the catalysts. The mineralization values evaluated from the Total Organic Carbon contents were also high for all of the catalysts, ranging from 96–98% after 120 min of irradiation (Table S1). It should be noted that the incorporation of the carbon material in the catalysts improved the conversion of the dye, indicating a strong impact of the carbon additive on the degradation (Figure 4 and Figure S5). This is in agreement with data reported in the literature for BWO and BWO/carbon catalysts using other carbon additives (e.g., activated carbons, graphene, carbon quantum-dots) [23,24,25,27]. The beneficial effect of the carbon additive was more pronounced at short irradiation times (the first 30–60 min), suggesting that the carbon additives accelerated the degradation reaction (Figure 4B). More interestingly, the photocatalysts prepared with 5 wt.% of carbon additive still showed a good catalytic activity, some of them outperforming the bare semiconductor (Figure 4 and Figure S5). Increasing the amount of carbon additive provoked a decrease in the degradation rate of all the samples, although the total conversion after 2 h of irradiation was still near 100%. For instance, after 15 min the conversion obtained by the bare semiconductor was 3 to 4 times lower (ca. 13%) than of BWO/CS-2 and BWO/CL-2. Hence, the degradation is notably slower for the former; the irradiation time needed for achieving 50% of RhB conversion was also 1–2 times higher for BWO than for any of the BWO/carbon catalysts (Figure 4B). In contrast, differences in the irradiation time to reach ca. 95% conversion were not so large, between 85–115 min (Figure 4B), except for BWO/CS with the fastest degradation rate. The fact that low carbon contents give rise to faster photocatalytic degradation rates can be attributed to the strong shielding effect of the carbon matrix observed in the catalysts with 5 wt.% of carbon additive (Figure 3).

It should also be pointed out that the photocatalytic degradation rate was particularly accelerated in the case of the catalysts prepared with carbons CS and CL. Indeed, the degradation rate was faster for BWO/CS, regardless the amount of carbon. Since all the catalysts displayed quite similar physicochemical features (Table 1) in terms of surface pH, porosity and crystallinity, this behavior is attributed to the differences in the composition of the carbon additive. In this regard, carbon CS and CL are characterized by a marked acidic nature compared to carbon CB, as inferred from ammonia chemisorption and surface pH values (Table 1). They also exhibit a high oxygen content (e.g., 24.8, 12.8 and 2.1 wt.% oxygen for CS, CL and CB respectively). A clear correlation is observed with the amount of oxygen functionalities and the acidity of the carbon materials and the improved photocatalytic response of the BWO/carbon photocatalysts.

The porosity of the catalysts has also been reported to play a role in photodegradation reactions [17,21,23]. As discussed above, the incorporation of the carbon additives provoked a slight increase in the micropore volume of samples prepared using carbons CB and CL, regardless of the amount of carbon. However, the most performing photocatalyst (both in terms of conversion yield, rate and mineralization) was the one prepared using carbon CS with a low porosity (Table 1). Thus, it appears that the effect of the composition and the acidic/basic nature of the carbon additives would predominate over their porosity.

Furthermore, the BWO/carbon catalysts showed good stability after 2 h of irradiation in aqueous dispersions, with no modifications observed in the XRD patterns of the catalysts (results not shown) or the composition of the carbon additive as shown by XPS (Table S2).

The analysis of the solution by UV-Vis spectrophotometry (Figure 4A) revealed the appearance of a hypsochromic shift (ca. 15–60 nm shift) in the spectra of RhB for all the catalysts, characteristic of the stepwise formation of N-deethylated subproducts [42]. This behavior was more pronounced for the catalyst prepared using carbons CL and CS. The formation of the N-deethylated intermediates was further confirmed by HPLC analysis (Figure S6); their concentration almost disappeared at long irradiation times (above 90 min), confirming that they are further decomposed during the photocatalytic reaction. The high mineralization values evaluated by TOC (from 96–98%, Table S1) after several hours of irradiation also supported this fact. Additionally, the decolorization of the solutions started to be noticed at long irradiation times, indicating the occurrence of a second mechanism accounting for the degradation of the N-deethylated chromophore ring [43]. Otherwise, the reaction would terminate with the formation of the fully N-ethylated intermediate, rendering low photocatalytic yields and mineralization values.

The predominance of the degradation route via the successive deethylation of the alkylamine moiety at short times is expected considering the overall acidic nature of the catalysts. In our experimental conditions RhB is a zwitterion with negative (carboxylic) and positive (diethylamino) charges in the auxochrome groups. Since our catalysts are mainly negatively charged (Table 1), RhB is preferentially adsorbed in the catalyst’s surface by electrostatic interactions with the diethylamino groups. The interactions are stronger for samples BWO/CL and BWO/CS due to their higher acidity (Table 1), which explains their better photocatalytic performance and faster degradation rates. A similar behavior has been reported for the degradation of RhB using other semiconductors (e.g., WO_3_, TiO_2_, silica-titania mixtures) with predominance of the successive deethylation mechanism favored in acidic photocatalysts [44,45] over the chromophore cleavage in basic ones [46].

**Figure 5:**
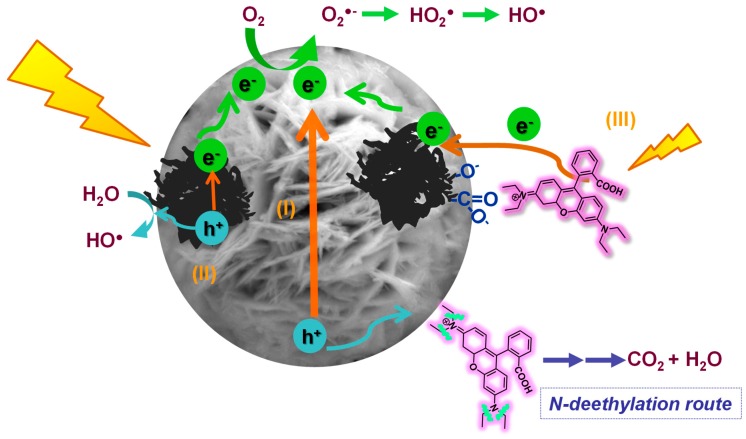
Schematic representation of the proposed photocatalytic mechanism for the degradation RhB using Bi_2_WO_6_/carbon catalysts under simulated solar light irradiation. Photocatalytic pathways via excitation of the semiconductor (I) and the carbon additive (II), and photosensitization (III).

RhB photodegradation may also proceed via a photosensitization mechanism since the dye itself absorbs in the range 460–600 nm as shown in Figure 4a. Thus, electrons resulting from the RhB self-photosensitization under visible light irradiation can be transferred to the carbon additive or to the conduction band of the semiconductor, contributing to the degradation of the dye [19,27]. In the absence of a catalyst, the photosensitization route does not have a large impact on the degradation of the dye. However, this could be occurring for the BiWO/carbon catalysts, especially for BWO/HC and BWO/CL where the injection of electrons from the photoexcited dye to the surface of the catalyst is favored by the stronger interactions between RhB and the catalyst surface (Figure 5) [47].

Another possibility is the separation of the photogenerated charge carriers (electrons and holes) through their reaction with water molecules co-confined in the pores of the catalysts, leading to the formation of reactive oxygen species (ROS). The ability of the BWO/carbon catalysts to transfer the photogenerated charge carriers was confirmed by electron spin resonance spectroscopy using a nitrone spin trapping agent (Figure S7). Data has confirmed a higher amount of hydroxyl radicals formed upon irradiation of aqueous suspensions of BWO/carbon catalysts under visible light. Additionally, it should be mentioned that some of the carbon additives used in this work (e.g., carbons CB and CS) have also shown the capacity themselves to photogenerate ROS upon illumination [29]. Thus, deethylation of RhB would occur by the attack of the hydroxyl radicals formed.

### 3.3. Evolution of the Toxicity of the Solutions During the Photocatalytic Runs

We studied the evolution of the toxicity of the solutions of RhB treated by the photocatalytic process using Bi_2_WO_6_/carbon materials with different carbon amounts in their composition, with the irradiation time. Unlike TOC measurements, the toxicity test is sensitive to the response of all the compounds present in the solution (e.g., RhB and its degradation intermediates), thus providing a quantification of the toxicity of the solution after the photocatalytic treatment. Since the luminescence of *V. Fischeri* is proportional to the metabolic activity of the bacterial population, the inhibition corresponds to lethal or sub-lethal response when it is exposed to a chemical agent; hence, a decrease in the luminescence signal indicates a higher toxicity of the solution and vice versa. This is important since the presence of heteroatoms (e.g., chlorine) can interfere the determination of TOC measurements; this is the case in the degradation of RhB, which presents nitrogen and chlorine atoms in its formula.

The luminescence inhibition was recorded after 5 and 15 min of exposure time of *V. Fischeri* bacteria to the solutions before and during the photocatalytic treatment. The toxicity of the initial RhB solution was also evaluated, rendering an inhibition of 77–81% that corresponds to a high toxicity of the initial solution. Indeed, the median effective concentration parameter (EC_50_) of Rhodamine B was ca. 2.5 ppm (Figure S8). This is a common toxicological descriptor for acute environmental hazard that refers to the concentration of a substance which results in 50 percent reduction of the bacteria luminescence. However, EC-50 values cannot be estimated in complex mixtures (i.e., during the photocatalytic reaction), thus toxicity was reported as the luminescence inhibition after exposure of the bacteria to the solution for samples with 45% dilution.

**Figure 6:**
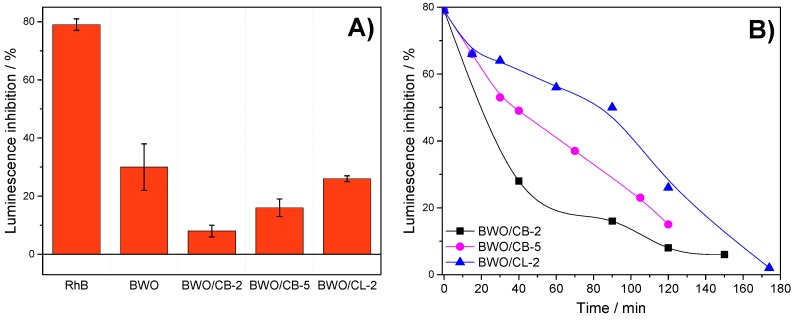
(**A**) Luminescence inhibition of BWO, BWO/CB-2, BWO/CB-5, and BWO/CL-2 photocatalysts after 120 min of irradiation, and response of the initial concentration of RhB (10 ppm) in solution; (**B**) Effect of the photocatalytic degradation using different BWO/carbon catalysts on the evolution of the bacteria luminescence inhibition with the irradiation time of aqueous solutions after an exposure time of 15 min.

Figure 6 presents the data obtained after 15 min exposure of the bacteria (the curves recorded after a 5 min exposure time followed similar trends) to the solutions irradiated for 2 h. Compared to the response measured for the initial solution of Rhodamine B, a sharp drop in the luminescence inhibition was observed for all the catalysts. This indicates that the treated solutions are less toxic than the initial one, despite certain catalysts do not achieve full conversion of the dye. It also suggests that the toxicity of the intermediate derivatives remaining in the solution (e.g., N- Cl- containing compounds) after the photocatalytic treatment is lower than that of the dye itself. It is also important to remark that the toxicity of the solution after the photolysis (Table S1) is expected to be very high (estimated luminescence inhibition of 68%) based on the concentration of RhB remaining in the solution, Figure S8.

Comparatively, lower values of toxicity were obtained for the BWO/carbon catalysts than for the bare semiconductor after 2 h or irradiation. This is in agreement with the amount of RhB measured in the solution after 120 min irradiation of BWO, and thus the lower conversion of the dye (ca. 89%) compared with BWO/carbon photocatalysts (ca. 98, 95, and 96% for BWO/CB-2, BWO/CB-5 and BWO/CL-2, respectively). Indeed, it was necessary to increase the irradiation time to 3 and 4 h for BWO to obtain the full conversion of the dye (Figure 4) and a luminescence inhibition similar to that measured for the BWO/carbon catalysts. This is also in agreement with the increased photodegradation rate of the catalysts incorporating carbon additives, as discussed above.

For the least performing catalysts (samples BWO/CL-2, BWO/CB-2 and BWO/CB-5), we recorded the evolution of the toxicity with the irradiation time. In the case of sample BWO/CB-2, the curve showed a sharp decrease in the luminescence inhibition in the first 30–60 min of irradiation time, in agreement with the fast kinetics observed for the photocatalytic reaction. When the amount of carbon was raised to 5 wt.% (sample BWO/CB-5), the luminescence inhibition reduction is still significant but follows a smoother trend with the irradiation time. For BWO/CL-2, the evolution of toxicity with the irradiation time is also slow in the first 60 min, and is somehow accelerated afterwards. We attribute this to the accumulation of degradation intermediates (short-chain carboxylic acids and N-deethylated compounds) at short irradiation times, that are further degraded at longer times. This correlates with the abundance of intermediates detected in solution by HPLC (Figure S6). The absence of luminescence inhibition peaks with the irradiation time suggests that the intermediates detected at short times most likely possess equal or smaller toxicity than RhB itself.

## 4. Conclusions

The photocatalytic performance of Bi_2_WO_6_/carbon catalysts prepared with increasing amounts of carbon additives towards the degradation of Rhodamine B has revealed the outstanding role of the physicochemical features of the carbon additive on the catalytic performance of the catalysts, and on the degradation pathway. Despite the strong shielding effect of carbon materials (decreasing a fraction of light that reaches the photoactive sites in the catalyst), Bi_2_WO_6_/carbon catalysis incorporating up to 5 wt.% of carbon material displayed a faster photodegradation rate than the bare semiconductor under simulated solar light. This effect was independent of the physicochemical properties of the carbon additive, although it was more pronounced for the catalysts incorporating carbons of acidic character with high oxygen content. The accelerated photodegradation evidenced that the presence of a carbon phase in the Bi_2_WO_6_/carbon catalysts favors the separation of the photogenerated charge carriers, leading to charge transfer reactions (direct or indirect). These may involve direct hole oxidation, due to the proximity of RhB adsorbed in the catalyst surface, and radical mediated mechanisms formed upon BWO/carbon-photon and carbon-photon interactions (mainly hydroxyl radicals). Our data has shown that the photocatalytic performance is dominated by surface interactions between the pollutant and the catalysts, with a low impact of the porosity. The overall acidity of the Bi_2_WO_6_/carbon catalysts controls the degradation pathway that proceeds via coupled mechanisms. Although they can take place simultaneously, our data suggest rather a subsequent mechanism with the deethylation mechanism dominates at short times (due to the preferential adsorption of the dye through the alkylamine group in the acidic catalysts), over the cleavage of the chromophore at long irradiation times, causing the decolorization of the solution. The degradation mechanism follows a similar pattern for the BWO/carbon composites, indicating that the effect of the carbon additive is linked to the kinetics of the photooxidation reaction rather than to a mechanistic change. Based on the conversion and high mineralization values, the presence of the carbon additive accelerates the conversion rate of the dye and its degradation intermediates, rendering a low toxicity after shorter irradiation periods. Furthermore, as the production costs of carbon materials are very low, the impact in the cost of the photocatalysts can be significantly reduced, favoring an eventual upscale of the photocatalytic process. Further studies on the optimization of the carbon additive and stability studies of the catalysts after long irradiation periods are still needed.

## Figures and Tables

**Table 1:** Main textural and physicochemical parameters of the BWO/carbon photocatalysts and the carbon additives.

Sample	S_BET_ (m^2^/g)	V_PORES_* (cm^3^/g)	W_0_ (cm^3^/g)	Surface pH	Acid Sites (mmol/g)
**BWO**	33	0.086	0.010	4.2	0.033
**BWO/CS-2**	41	0.106	0.013	4.6	0.079
**BWO/CL-2**	43	0.123	0.014	4.5	0.095
**BWO/CB-2**	41	0.100	0.011	4.8	0.036
**BWO/CS-5**	53	0.088	0.014	3.6	0.087
**BWO/CL-5**	46	0.106	0.016	3.5	0.211
**BWO/CB-5**	40	0.105	0.017	5.2	-
**CS**	10	0.020	0.01	4.3	0.480
**CL**	1280	1.060	0.31	3.6	0.406
**CB**	1031	0.520	0.320	9.0	0.010
* evaluated at p/p_0_ ≈ 0.99					

## Supplementary Materials

The following are available online at http://www.mdpi.com/1996-1944/12/3/385/s1, Table S1. Rhodamine B conversion and Total Organic Carbon (TOC) values upon 2 h of irradiation of the studied catalysts; Figure S1. Emission spectrum of the lamp used in the photocatalytic experiments; Table S2. Surface concentration of carbon species obtained by fitting the C 1s core level peak of the XPS spectra of composites BWO/CL-2 and BWO/CS-2 as received (fresh) and after irradiation of an aqueous dispersion to explore the stability of the carbon component. Figure S2. SEM images of the carbon materials used as additives (samples CS, CB and CL); Figure S3. X-ray diffraction patterns of (A) BWO and BWO/carbon catalysts and (B) the carbon materials used as additives. Diffractograms have been shifted for clarity; Figure S4. Nitrogen adsorption/desorption isotherms at 77 K of the studied photocatalysts; Figure S5. Rhodamine B conversion upon exposure to simulated solar light of the catalysts with 2 (A) and 5 wt.% (B) of carbon additive; Figure S6. Evolution of RhB photooxidation intermediates (Rhodamine, R; N-ethylrhodamine, ER; N,N-diethylrhodamine, DR; N-ethyl-N’’-ethylrhodamine, EER; N,N-diethyl-N’’-ethylrhodamine, DER) upon irradiation of photocatalysts BWO, BWO/CS-2 and BWO/CL-2; Figure S7. (left) Characteristic ESR signals corresponding to DMPO-OH adducts obtained upon 20 min of irradiation of an aqueous suspension of BWO and BWO/carbon photocatalysts in the presence of DMPO as trapping agent; (right) Quantification of O radical species by integration of the second peak in the 1:2:2:1 quartet profile of the DMPO-OH adducts of selected photocatalysts; Figure S8. Luminescence inhibition of Vibrio Fischeri bacteria upon exposure to Rhodamine B aqueous solutions for 15 min. The toxicological parameter EC50 determined as the concentration of RhB for a 50% inhibition was ca. 2.5 ppm.
