# Supplementary material for: Insights on the Use of Carbon Additives as Promoters of the Visible-Light Photocatalytic Activity of Bi2WO6
Source: Materials (Basel). 2019 Jan 26;12(3):385. doi: 10.3390/ma12030385 (PMC6384959; doi:10.3390/ma12030385)
Supplement: Supplementary file 1 [file materials-12-00385-s001.pdf]

Supporting Information File

Insights on the use of Carbon Additives as Promoters of the Visible-Light Photocatalytic Activity of Bi_2WO_6

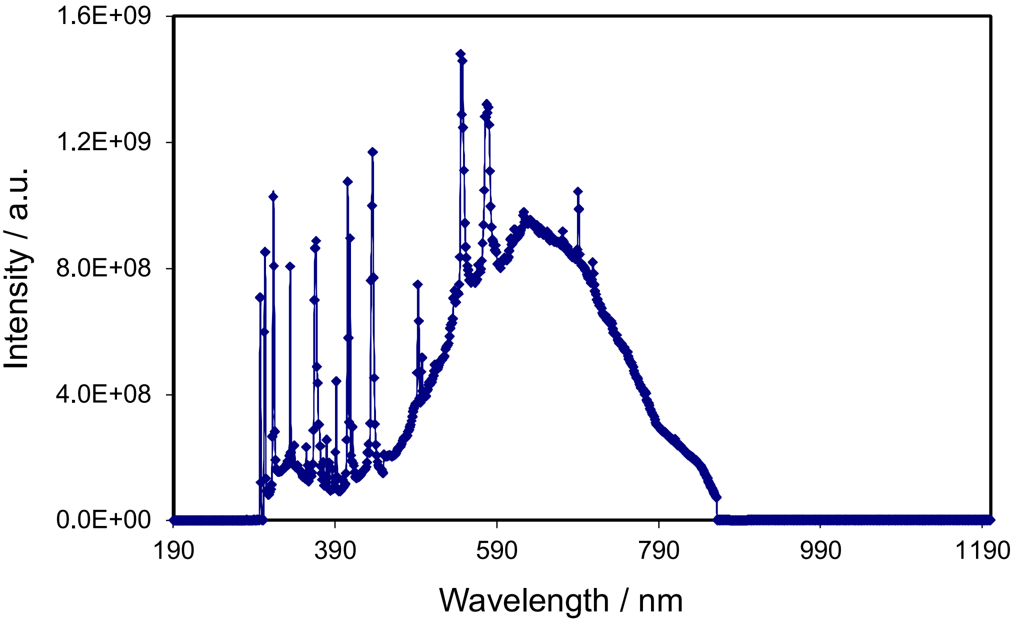

Figure S1. Emission spectrum of the lamp used in the photocatalytic experiments.

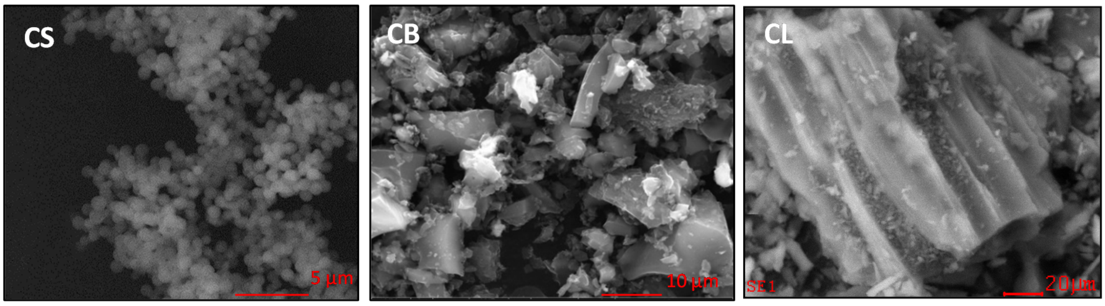

Figure S2. SEM images of the carbon materials used as additives (samples CS, CB and CL).

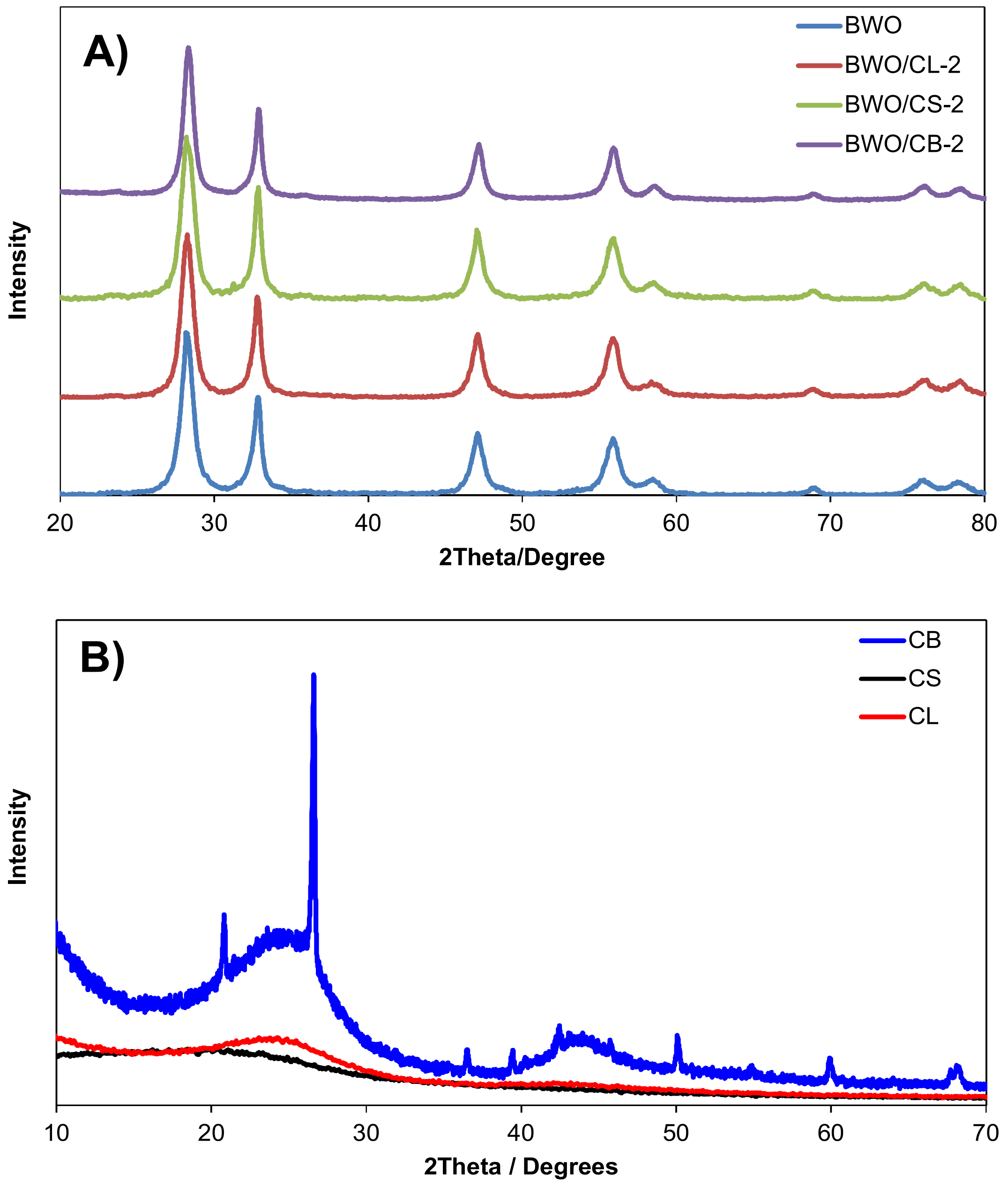

Figure S3. X-ray diffraction patterns of A) BWO and BWO/carbon catalysts and B) the carbon materials used as additives. Diffractograms have been shifted for clarity.

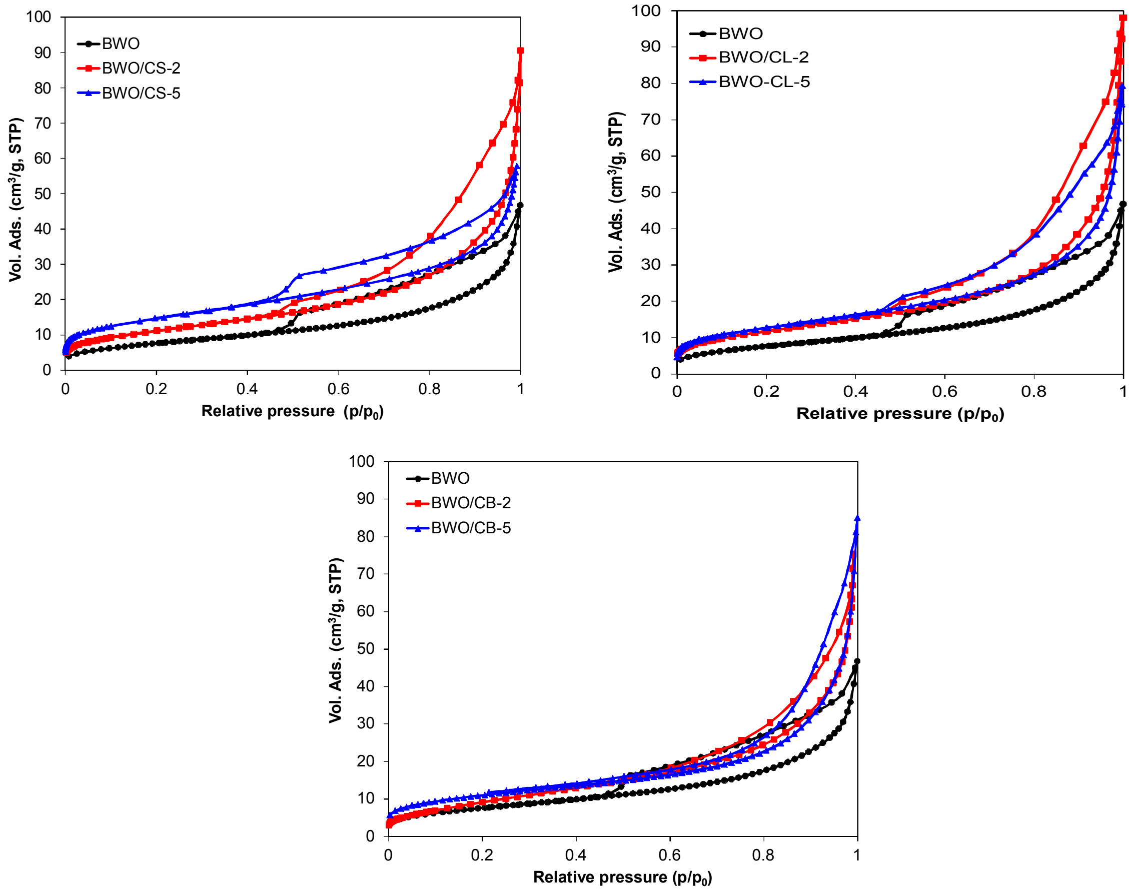

Figure S4. Nitrogen adsorption/desorption isotherms at 77 K of the studied photocatalysts.

Table S1. Rhodamine B conversion and Total Organic Carbon (TOC) values upon 2 h of irradiation of the studied catalysts.

	**Conversion at 120 min**	**TOC initial**	**TOC final**	**Mineralization ([1-(Final TOC/InitialTOC)])**
	**(%)**	**(mgC/L)**	**(mgC/L)**	**(%)**
**RhB Photolysis**	6	6.8	6.5	4.4
**BWO**	89	7.4*	0.3	95.9
**BWO/CS-2**	99	6.3	0.1	98.4
**BWO/CL-2**	96	8.3	0.3	96.4
**BWO/CB-2**	98	6.8	-	-
**BWO/CS-5**	99	8.3	0.14	98.3
**BWO/CL-5**	94	7.4	0.42	94.3
**BWO/CB-5**	95	6.3	-	-

*Measured at 180 min

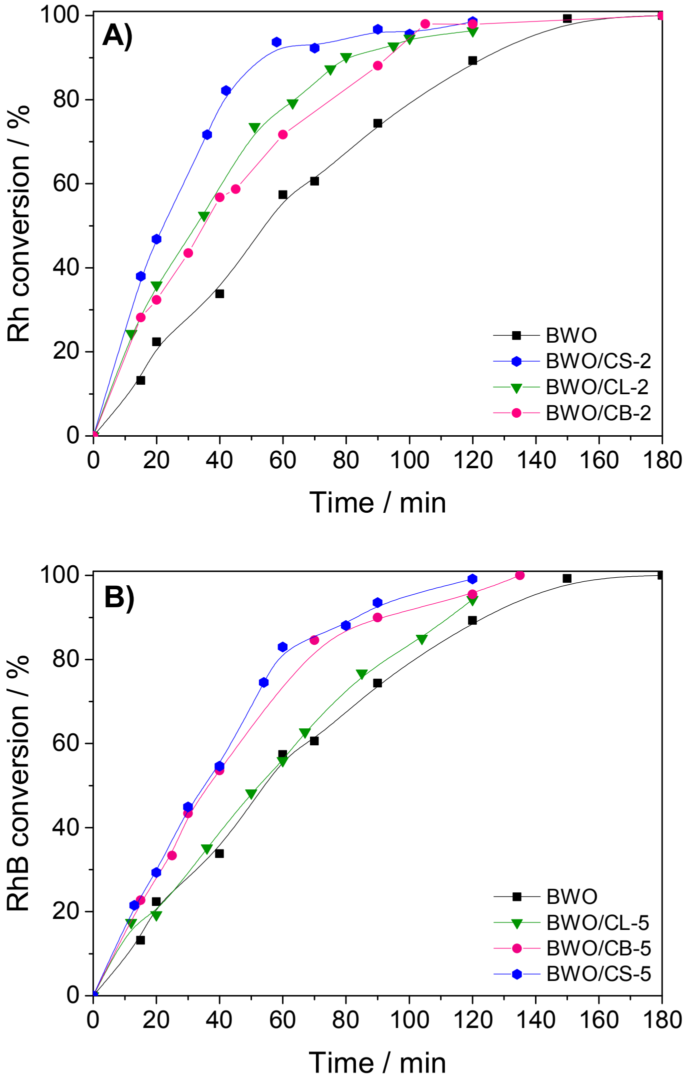

Figure S5. Rhodamine B conversion upon exposure to simulated solar light of the catalysts with 2 (A) and 5 wt. % (B) of carbon additive.

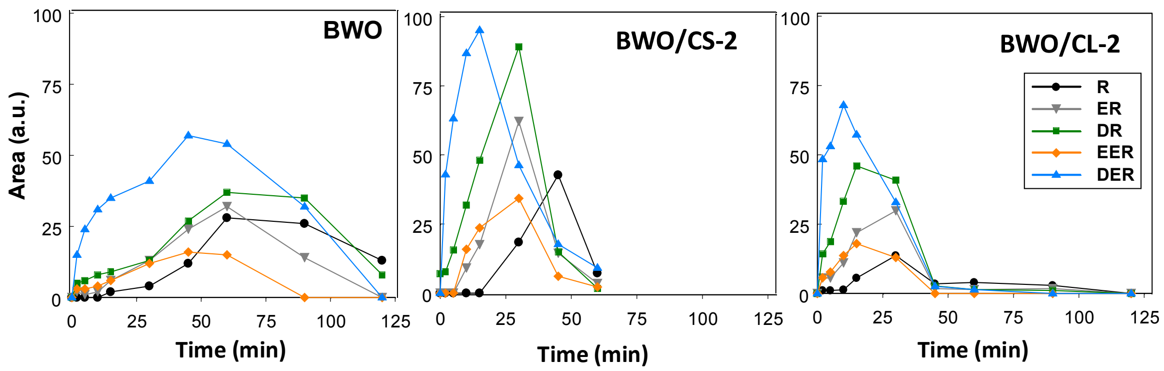

Figure S6. Evolution of RhB photooxidation intermediates (Rhodamine, R; N-ethylrhodamine, ER; N,N-diethylrhodamine, DR; N-ethyl-N''-ethylrhodamine, EER; N,N-diethyl-N''-ethylrhodamine, DER) upon irradiation of photocatalysts BWO, BWO/CS-2 and BWO/CL-2.

Table S2. Surface concentration of carbon species obtained by fitting the C 1*s* core level peak of the XPS spectra of composites BWO/CL-2 and BWO/CS-2 as received (fresh) and after irradiation of an aqueous dispersion to explore the stability of the carbon component.

Bond assignement (energy, eV)	Fresh	Irradiated
**BWO/CL-2**		
C–C (graphitic carbon - 284.6 eV)	59.3	62.7
C–O (phenolic, alcoholic, etheric - 286.1 eV)	13.8	13.1
C=O (carbonyl or quinone - 287.1 eV)	17.7	18.2
O–C=O (carboxyl or ester - 288.7 eV)	5.8	6.0
π–π* (291.0 eV)	3.4	3.5
**BWO/CS-2**		
C–C (graphitic carbon - 284.6 eV)	66.5	65.1
C–O (phenolic, alcoholic, etheric - 286.1 eV)	15.9	17.5
C=O (carbonyl or quinone - 287.1 eV)	6.8	7.8
O–C=O (carboxyl or ester - 288.7 eV)	7.5	8.2
π–π* (291.0 eV)	3.3	1.3

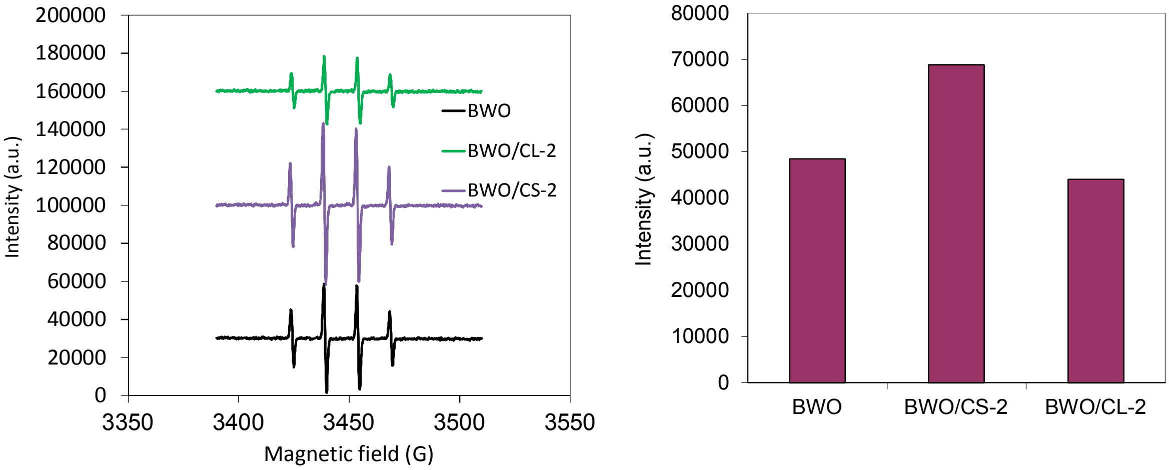

Figure S7. (left) Characteristic ESR signals corresponding to DMPO-OH adducts obtained upon 20 min of irradiation of an aqueous suspension of BWO and BWO/carbon photocatalysts in the presence of DMPO as trapping agent; (right) Quantification of O-radical species by integration of the second peak in the 1:2:2:1 quartet profile of the DMPO-OH adducts of selected photocatalysts.

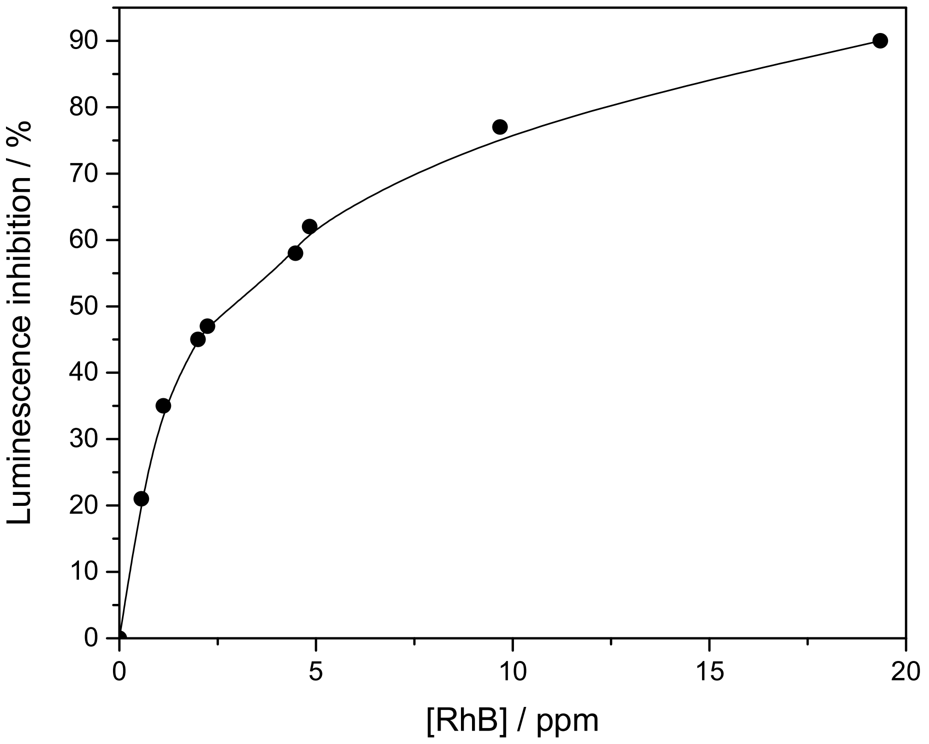

Figure S8. Luminescence inhibition of *Vibrio Fischeri* bacteria upon exposure to Rhodamine B aqueous solutions for 15 min. The toxicological parameter EC_{50} determined as the concentration of RhB for a 50% inhibition was ca. 2.5 ppm.
